# Clinical Impact of Polygenic Risk Score for Breast Cancer Risk Prediction in 382 Individuals with Hereditary Breast and Ovarian Cancer Syndrome

**DOI:** 10.3390/cancers15153938

**Published:** 2023-08-02

**Authors:** Sarah Stiller, Stephan Drukewitz, Kathleen Lehmann, Julia Hentschel, Vincent Strehlow

**Affiliations:** 1 Institute of Human Genetics, University of Leipzig Medical Center, 04103 Leipzig, Germany; 2 Core Unit for Molecular Tumor Diagnostics (CMTD), National Center for Tumor Diseases (NCT), Partner Site Dresden, 01307 Dresden, Germany

**Keywords:** breast cancer, risk prediction, polygenic risk score, genetic counselling, hereditary breast and ovarian cancer syndrome

## Abstract

**Simple Summary:**

Breast cancer (BC) is the major cause of cancer-related deaths in women worldwide. In addition to genetic diagnostics for variants in high-risk genes, there is a need for better risk stratification to target high-risk individuals. The polygenic risk score (PRS) has emerged as a valuable addition to help sorting women into different risk categories for BC development. This study aimed to evaluate the impact of adding a PRS, based on 313 genetic variants, to standard genetic testing for 382 German women with BC or a family history of the disease. By incorporating the PRS into risk prediction models, meaningful changes in 10-year risks were observed in 13.6% of individuals. Additionally, the inclusion of the PRS led to clinically significant changes in prevention recommendations for 12.0% of cases, supporting the use of the PRS for BC risk assessment in genetic counselling.

**Abstract:**

Single nucleotide polymorphisms are currently not considered in breast cancer (BC) risk predictions used in daily practice of genetic counselling and clinical management of familial BC in Germany. This study aimed to assess the clinical value of incorporating a 313-variant-based polygenic risk score (PRS) into BC risk calculations in a cohort of German women with suspected hereditary breast and ovarian cancer syndrome (HBOC). Data from 382 individuals seeking counselling for HBOC were analysed. Risk calculations were performed using the Breast and Ovarian Analysis of Disease Incidence and Carrier Estimation Algorithm with and without the inclusion of the PRS. Changes in risk predictions and their impact on clinical management were evaluated. The PRS led to changes in risk stratification based on 10-year risk calculations in 13.6% of individuals. Furthermore, the inclusion of the PRS in BC risk predictions resulted in clinically significant changes in 12.0% of cases, impacting the prevention recommendations established by the German Consortium for Hereditary Breast and Ovarian Cancer. These findings support the implementation of the PRS in genetic counselling for personalized BC risk assessment.

## 1. Introduction

Breast Cancer (BC) is the most prevalent form of cancer in women and the leading cause of cancer-related death among women worldwide [1]. To facilitate early detection of BC, improve chances of recovery, and decrease mortality rates, screening programs have been established [2,3]. However, current screening strategies, such as clinical examinations and mammograms, are vulnerable to overdiagnosis and overtreatment [4,5].

Personalized risk estimations of BC could help to improve prevention and screening programs by identifying women in risk categories who are most likely to benefit [6].

A positive family history is one of the primary risk factors for developing the disease [7]. Approximately 30% of all women with BC in Germany show a positive family history and fulfil the inclusion criteria of the German Consortium for Hereditary Breast and Ovarian Cancer (GC-HBOC), making them eligible for extensive screening measures [3,8].

The genetic susceptibility to BC is determined by rare high-penetrance variants in *BRCA1*, *BRCA2*, *PALB2*, and *TP53* [9,10,11], likewise rare moderate-risk variants (e.g., in *ATM*, *CHEK2,* and *RAD51C*) [12], as well as common low-risk variants, predominantly single nucleotide polymorphisms (SNPs) [13,14]. According to genome-wide association studies, each individual SNP can either slightly increase or decrease the risk of developing BC. When summarizing all SNPs in a polygenic risk score (PRS), the cumulative risk can be substantial [15,16]. Thus, the PRS can aid in stratifying women into different risk categories of developing BC. Moreover, the BC risk of women carrying a pathogenic variant (PV) in *ATM*, *BRCA1/2*, *CHEK2*, or *PALB2* can also be refined using the PRS [17,18].

Belgium, France, Israel, Italy, the United Kingdom, and Spain are comparing personalized, risk-stratified BC screening to standard screening as part of the ongoing international MyPeBS study [19]. In Germany, however, the use of the PRS for risk prediction has not yet been incorporated into the daily practice of genetic counselling for familial BC. 

The risk prediction is currently estimated by risk prediction algorithms [20] based on family history such as the Breast and Ovarian Analysis of Disease Incidence and Carrier Estimation Algorithm (BOADICEA) [21], which has been proven to identify women at high risk more accurately than other models [22] and has been prospectively validated [23,24]. Multiple studies presented that by combining the PRS with other known risk factors for BC risk stratification, discriminatory power between BC cases and controls can be improved [25,26,27,28]. 

Risk prediction tools such as the CanRisk [29] webtool using BOADICEA and a PRS based on 313 variants have recently been made accessible for healthcare professionals [30]. BOADICEA has also been updated to incorporate alternative PRSs and can be readily adapted to different PRSs in a manner that maintains consistency of the model [31].

In the present work, we examined the clinical value of the PRS for BC risk prediction based on personal medical history, family history, and PV carrier status in a cohort of 382 German women with suspected hereditary breast and ovarian cancer syndrome (HBOC). 

We examined the extent to which the inclusion of the PRS leads to a relevant change in BC risk predictions. The impact of the PRS on BC risk prediction was then assessed by determining the possible change in clinical management, as stated in the guideline by the GC-HBOC. The guideline defines a specific group of women who are entitled to enrol in an intensified BC surveillance program if their calculated 10-year risk of developing BC is above 5% [8]. 

## 2. Materials and Methods

### 2.1. Study Cohort

We collected data from women from families with suspected HBOC and from families in which a PV in the causative genes for HBOC is already known from December 2020 to January 2023. All participants sought counselling for HBOC at the Institute of Human Genetics of the University of Leipzig, Germany, which is part of the Centre for Hereditary Breast and Ovarian Cancer Leipzig. A total of 382 women met the following eligibility criteria for this study:

(1) Availability of genotyping data;
(2) Availability of personal medical information (e.g., cancer diagnosis);
(3) Available information on family history (e.g., family members with a cancer diagnosis);
(4) Age range of 18 to 69 years;
(5) The presence of a PV for which a risk calculation using CanRisk was possible in case of a positive carrier status.

Women with BC or OC were included if they met additional criteria:

(1) Available information on tumour pathology;
(2) Available clinical data such as age of first onset of disease;
(3) Unilaterality in women with BC.

For individuals with unilateral BC, the risk of developing a contralateral tumour was calculated. Women with bilateral BC, ductal carcinoma in situ (DCIS), or pancreatic cancer could not be considered as they did not meet the requirements for BOADICEA computation [32]. All participants provided informed consent for their data to be used for research purposes. All individuals were anonymized.

### 2.2. Pedigree Collection and Initial Risk Assessment

The pedigrees included in this work were generated using the PhenoTips webtool [33] during the initial consultation. All participants’ pedigrees include at least three generations. The following data were available for each individual in the pedigrees:

(1) Life status;
(2) Year of birth and current age;
(3) Cancer status.

The age of first onset of disease was known for all index patients.

All participants underwent an additional risk assessment using the checklist of The German Cancer Society [34,35]. The checklist is based on associated cases of HBOC in the maternal and paternal line, which are included with either single, double, or triple weighting while also considering the age of first onset of disease and the hormone receptor status in BC cases. Individuals with a score of ≥3 are considered to have a risk of carrying a PV of at least 10% and are thus eligible for genetic testing [36,37,38].

### 2.3. Molecular Genetics

All women from families with suspected HBOC underwent panel diagnostics including PRS testing. Some of the women, in whom a PV was known in the family, received the targeted testing within the framework of panel diagnostics including PRS testing, the rest received targeted testing by means of Sanger sequencing and additional PRS testing. 

Genomic DNA was extracted from whole blood using MagCore Kit 101 and MagCore^®^ instrument (RBC Bioscience, New Taipei City, Taiwan). DNA concentration was measured using NanoDrop™ 2000 (Thermo Scientific™, Waltham, MA, USA) and Qubit (Thermo Scientific™,Waltham, MA, USA). Next generation sequencing (NGS) after sample preparation using Twist Library Preparation EF Kit Twist Library Preparation EF Kit1, 2.0, and Twist Universal Adapter System—TruSeq Compatible, 96 Samples Plate A-D, enrichment using Twist Custom Panel, design name: Cancer_PRS_HUGV6; Twist Design ID: TE-96674869 (Twist Bioscience, South San Francisco, CA, USA), and sample identification using the Nimagen RC-PCR assay. Sequencing was conducted on a NextSeq500/550 Mid Output v2.5 kit (Illumina, San Diego, CA, USA; Sequencer: Illumina NextSeq550 (Illumina, San Diego, CA, USA). Mean coverage was at least 300×, and all target regions were covered 20×. Single nucleotide variants (SNVs) as well as copy number variants (CNVs) were detected within this setting. This analysis can be established in all labs familiar with NGS settings.

### 2.4. Variant Classification and Panel Sequencing

Analysis of the raw data was performed using the software Varfeed (Limbus, Rostock, Germany), and the variants (SNVs and CNVs) were annotated using the software Varvis (Limbus, Rostock, Germany). All variants were described in regard to GRCh37 (NM_000492.4) and classified according to the latest ACMG criteria [39]. The databases ClinVar [40], HGMD [41], and HerediCare [42] were used for classification based on the following considerations: gene and variant attributes, frequency in the general population (gnomAD [43]), (assumed) effect on protein function, in silico prediction tools (mainly CADD [44], SpliceAI-lookup [45]), conservation, and phenotype.

### 2.5. Targeted Sequencing for PRS Calculation

Raw reads were quality checked using fastqc [46], and remaining adapter sequences and bad quality data were removed using trimmomatic [47]. Processed data was aligned to hg19 using minimap2 [48], visual duplicates were marked with samtools [49]. Haplotypes for the BCAC-313 PRS model were called using freebayes, and positions with a coverage of <20× or conflicting haplotype signal were imputed using twice the allele frequency described in the BCAC-313 model [50]. The resulting haplotypes were used as an input to calculate the normalized z-score via the CanRisk API [29]. Resulting z-scores were combined with the related BOADICEA file to calculate the 5-year, 10-year, and lifetime risk for each participant.

### 2.6. Statistical Testing

The PRS values of the study cohort were tested for normal distribution using the Shapiro–Wilk test from the scipy package [51].

The study cohort was compared to the BCAC-313 model [52] cohort using the mean (mu) and standard deviation (sigma) provided by CanRisk. PRS distribution of the BCAC-313 cohort was modelled using the numpy package, and the comparison between the cohorts was conducted using the individual t-test from the scipy package (Supplementary Figure S1). 

To test the effect of the PRS value on the 10-year risk calculation, the study cohort was filtered for women under the age of 50 without any known PVs in the 11 core genes, the cohort was divided in to high and low PRS individuals by comparison to the mean PRS value described for the BCAC-313 model. The effect of including or excluding the PRS value in the 10-year BC risk was calculated using the Mann–Whitney U test from the scipy package (Supplementary Figure S2).

## 3. Results

We included 382 women in our cohort. The mean age at time of evaluation was 45 years with an age range between 18 and 69 years. A total of 233 women (60.9%) were aged 18-49 years and 149 (39.1%) were aged 50 and older. At the time of analysis, 48.7% of women (186) had an invasive breast tumour, 7.9% (30) had ovarian cancer (OC), and 0.5% (2) were affected by both BC and OC. The average age at diagnosis for women affected by BC was 46 years with a range between 27 and 67 years. For OC cases, the mean age of diagnosis was 54 with a range between 22 and 67 years.

A total of 273 participants (71.5%) were evaluated for PVs in the 11 core genes *ATM*, *BARD1*, *BRCA1*, *BRCA2*, *CDH1*, *CHEK2*, *BRIP1*, *PALB2*, *RAD51C*, *RAD51D,* and *TP53* via a multigene panel diagnostic [53]. A total of 109 women (28.5%) received targeted genetic testing via Sanger sequencing.

A total of 206 of 218 cases affected by cancer received a multigene panel diagnostic, revealing 39 carriers of a PV (35.8% of all PV carriers). Twelve cancer patients received targeted genetic testing, leading to detection of a further nine PV carriers (8.3% of all PV carriers). Among the 164 healthy participants, multigene panel analysis for 67 of them (40.8%) revealed 17 carriers of a PV (15.6% of all PV carriers), while targeted genetic testing in the further 97 individuals identified 44 additional PV carriers (40.3% of all PV carriers) (Table 1).

**Figure 1:**
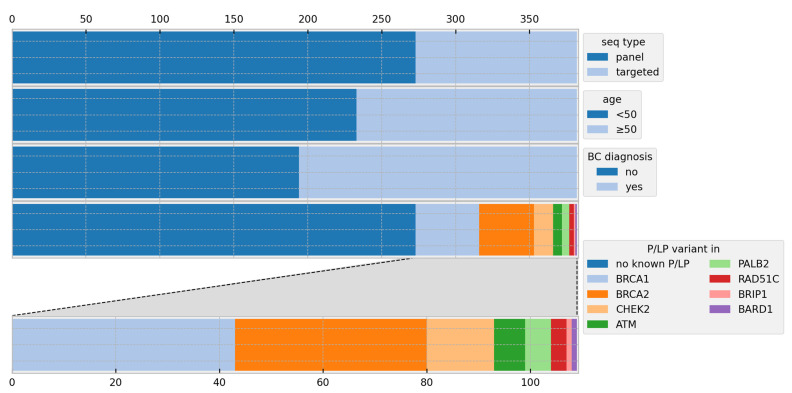
Cohort overview showing the proportions of participants regarding the sequencing methods, age distribution, breast cancer diagnosis, and pathogenic variants; BC = breast cancer, P/LP = pathogenic and likely pathogenic, and seq = sequencing.

Most participants were carriers of PVs in either *BRCA1* (11.3% of participants) or *BRCA2* (9.7%). Other PVs were found in *ATM* (1.6%), *BARD1* (0.3%), *BRIP1* (0.3%), *CHEK2* (3.4%), *PALB2* (1.3%), and *RAD51C* (0.8%). (Figure 1)

### 3.1. Risk Calculations

The PRS (z-score) in our cohort is normally distributed with a mean of 0.45 (SD = 1.02, Shapiro–Wilk test *p*-value = 0.79) (Supplementary Figure S1).

**Figure 2:**
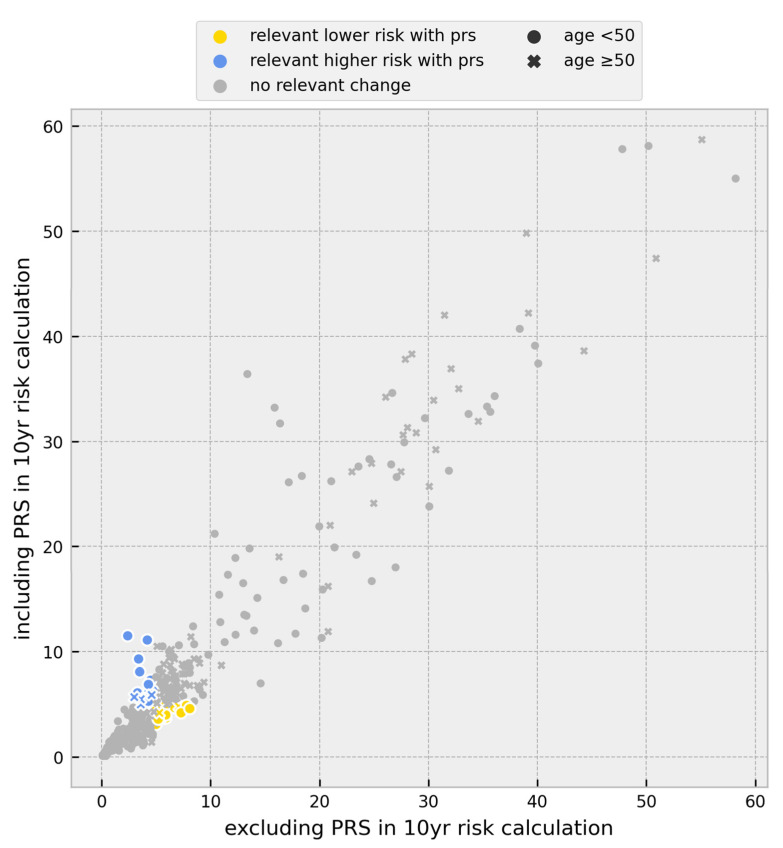
Scatter plot of the change in individual breast cancer 10-year risk after inclusion of the PRS for all probands. The initial breast cancer 10-year risk based on age, pedigree information, and PV status was plotted against breast cancer 10-year risk including the 313-variant PRS in addition to initial breast cancer 10-year risk. Individuals surpassing the 5% threshold after PRS inclusion are shown as blue symbols. Individuals falling below the 5% threshold after PRS inclusion are shown as yellow symbols; PRS = polygenic risk score, and PV = pathogenic variant.

Through the inclusion of the PRS, the threshold of 5% in 10-year risk calculations was observed to be either surpassed or subordinated in 13.6% of all individuals. Among them, 8.1% surpassed the 5% threshold, defining them as women at high risk of developing BC according to the GC-HBOC. The basis for establishing the threshold is that the 10-year risk of ≥5% is approximately double the value of a 50-year-old woman from the general population [8]. A total of 5.5% fell below the 5% threshold, indicating a relevantly lower risk (Figure 2). 

For 153 BC cases without any PVs, the inclusion of the PRS in 10-year risk calculations led to different risk stratification for 20.3%, with 11.1% surpassing the 5% threshold and 9.2% falling below. A total of 52.9% of cases exceeding the 5% threshold were under the age of 50.

**Figure 3:**
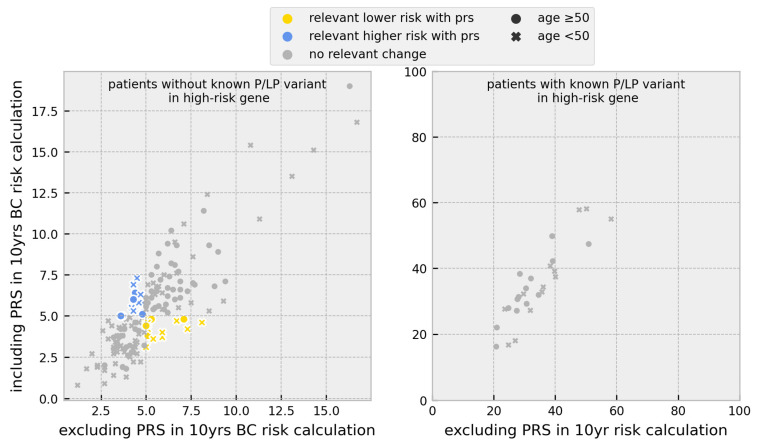
Scatter plot of the change in individual breast cancer 10-year risk after inclusion of the PRS for all BC cases separated by PV carrier status; BC = breast cancer, P/LP = pathogenic and likely pathogenic, PRS = polygenic risk score, and PV = pathogenic variant.

No changes in risk stratification after including the PRS were observed in the 27 BC cases with PVs in high-risk genes (*BRCA1*, *BRCA2*, and *PALB2*) (Figure 3).

The effect of including or excluding the PRS value in the 10-year BC risk calculation showed that individuals with lower PRS values than the mean described for the BCAC-313 model exhibit a significantly lower 10-year risk after incorporating the PRS into the analysis (*p*-value = 0.0011). In contrast, women with PRS values above the mean PRS value do not show a significantly higher 10-year risk (*p*-value = 0.31). However, it is important to note that for a specific individual, a higher PRS can still translate to a higher 10-year risk (Supplementary Figure S2).

### 3.2. Change in Prevention Management

The GC-HBOC considers risk calculations of the following groups for inclusion in the intensified breast cancer surveillance program [8,54,55]:

(1) Healthy women under the age of 50 with unremarkable predictive multigene panel diagnostic results;
(2) Healthy women under the age of 50 with unremarkable targeted genetic testing in moderate-risk genes;
(3) Ovarian cancer patients under the age of 50 with unremarkable multigene panel diagnostic results;
(4) Relatives of index patients with a PV detected by the multigene panel diagnostic. We did not include this group in our analyses.

If individuals included in one of these groups have a 10-year risk of ≥5% for developing BC, they are eligible for intensified screening to timely detect a potential breast tumour.

**Figure 4:**
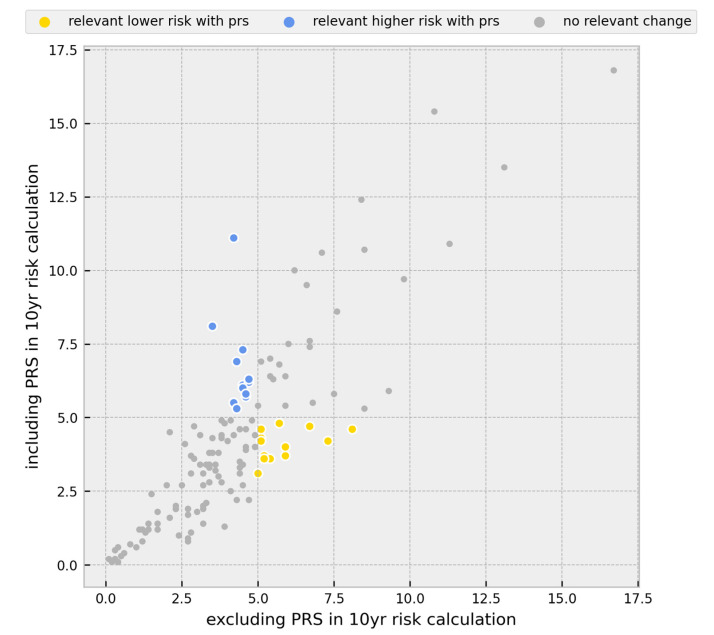
Scatter plot of the change in individual breast cancer 10-year risk after inclusion of the PRS for healthy women age < 50 with unremarkable predictive multigene panel diagnostic results, healthy women age < 50 with unremarkable targeted genetic testing in moderate-risk genes, and ovarian cancer patients age < 50 with unremarkable multigene panel diagnostic results. Individuals surpassing the 5%-risk threshold and consequent changes in clinical management are shown as blue dots. Individuals falling below the 5%-risk threshold and consequent changes in clinical management are shown as yellow dots; BC = breast cancer, and PRS = polygenic risk score.

A total of 30 participants in our cohort met the characteristics of the first group. By including the PRS in 10-year risk estimations, 16.7% of these participants either exceeded or fell below the 5% threshold, indicating a change in clinical management. There are 49 individuals that can be attributed to the second group with relevant changes in 10-year risk predictions observed in 10.2% of cases. For the four women attributed to the third group, there were no changes. Overall, 12.0% of participants that can be assigned to one of the groups would require a change in prevention management (Figure 4).

## 4. Discussion

In this study, we demonstrated the impact of the 313-variant-based PRS on BOADICEA-based BC risk calculations in a cohort of 382 women, resulting in changes of BC risk stratification in 13.6% of all participants. These variations in risk assessment might have significant implications, e.g., for making informed decisions regarding preventive surgeries. 

The majority of BC patients without a PV who have exceeded the 5% threshold after including the PRS in 10-year risk calculations are younger than 50 years (52.9%), indicating that this particular group would benefit from a more precise risk assessment due to the incorporation of the PRS. The association between PRS and BC risk decreasing with age was shown by Mavaddat et al. [13].

This is the first study conducted on the clinical application of the PRS in Germany. In a Dutch study, the impact of incorporating the PRS into risk calculations for 1331 non-*BRCA1/2* carriers was investigated regarding screening procedures aligned with Dutch IKNL [56], UK NICE [57], and US NCCN [58] BC screening guidelines. The results revealed clinically significant shifts in 32.4%, 36.0%, and 25.7% of individuals (with 30% BC lifetime risk cut-off levels based on the IKNL and NICE BC screening guidelines) [59].

In our cohort, the inclusion of the PRS in BC risk predictions resulted in clinically relevant shifts leading to changes in prevention recommendations established by the GC-HBOC in 12.0% of participants that can be assigned to one of the groups defined by the consortium. This presents a different patient stratification from current clinical practice, in which solely family history is included in risk prediction.

The current susceptibility SNPs account for around 44% of the familial relative risk associated with common low-risk variants [60]. Recent genome-wide association studies have discovered new BC susceptibility loci [61,62], leaving a more extensive PRS to be expected in the future. By incorporating an expanded PRS in BC risk prediction, more accurate risk stratification can be achieved, resulting in a higher percentage of women transitioning to different risk categories, and leading to improved screening measures.

Based on the considerable utility of the PRS, a potential application beyond HBOC families as part of general cancer screening should be discussed. A meta study was conducted to assess the cost effectiveness of implementing the PRS for three prevalent cancer types (prostate, colorectal, and breast cancer). Out of the ten studies analysed, eight demonstrated cost effectiveness in the utilization of the PRS [63]. However, further prospective studies with a larger cohort and case control studies are needed to quantify the effect of incorporation of the PRS in general screening among the general population programs for the health care system.

The strengths of this study include good representation of families that are seen in genetic counselling. All participants provided thorough family and personal medical history, ensuring comprehensive data collection. The utilization of BOADICEA as a well-validated and comprehensive risk model, allows for accurate risk predictions in a familial setting [22].

### Limitations

We had to assume the European ancestry based on the participants’ names. So far, the PRS is only validated for people of European ancestry [21], though some studies have indicated associations between a subset of the PRS and BC in Asian [64] and Latina/x/o [65] populations.

## 5. Conclusions

In summary, our findings support implementation of the PRS in genetic counselling, although it might present logistical challenges. By utilizing a reliable and comprehensive risk prediction model such as BOADICEA, pedigree-based family history, individual PRS, and molecular genetic analysis results can be combined easily, enabling the calculation of personalized BC risks, and therefore allowing for an improved clinical management in BC prevention.

## Figures and Tables

**Table 1:** Characteristics of cohort; BC=breast cancer, OC=ovarian cancer, and PV=pathogenic variant.

	Individuals
n	382
Age	
Mean	45
Range	18–69
Healthy	164
Affected	218
Affected by BC	186
Affected by OC	30
Affected by BC + OC	2
Age of onset of disease BC	
Mean	46
Range	27–67
Age of onset of disease OC	
Mean	54
Range	22–67
Array	
Multigene panel	273
Performed for healthy probands	67
Detected PVs	17
Performed for affected probands	206
Detected PVs	39
Sanger + PRS only	109
Performed for healthy probands	97
Detected PVs	44
Performed for affected probands	12
Detected PVs	9

## Data Availability

The data that support the findings of this study are available on request from the corresponding author.

## Supplementary Materials

The following supporting information can be downloaded at: https://www.mdpi.com/article/10.3390/cancers15153938/s1, Figure S1: The distribution of the PRS in our cohort and that of the UK cohort. The PRS is normally distributed; PRS = polygenic risk score, Figure S2: Scatter plot of the change in individual breast cancer 10-year risk after inclusion of the PRS for all BC cases under the age of 50. Individuals are separated into high and low PRS values compared to the mean PRS value described for the BCAC-313 model; BC = breast cancer, BCAC = Breast Cancer Association Consortium, PRS = polygenic risk score; PV = pathogenic variant

## References

[1] Sung H., Ferlay J., Siegel R.L., Laversanne M., Soerjomataram I., Jemal A., Bray F. (2021). Global Cancer Statistics 2020: GLOBOCAN Estimates of Incidence and Mortality Worldwide for 36 Cancers in 185 Countries. CA Cancer J. Clin..

[2] Quante A.S., Strahwald B., Fischer C., Kiechle M. (2018). Kiechle Individualized risk of breast cancer—How should it be calculated, evaluated and discussed?. Gynakologe.

[3] (2021). Deutsche Krebsgesellschaft. Deutsche Krebshilfe, and AWMF S3-Leitlinie Früherkennung, Diagnose, Therapie und Nachsorge des Mammakarzinoms, Version 4.4, Juni 2021. AWMF Registernummer: 032-045OL. https://www.leitlinienprogramm-onkologie.de/leitlinien/mammakarzinom/.

[4] Marmot M., Altman D.G., Cameron D.A., Dewar, Thompson S.G., Wilcox M. (2012). The benefits and harms of breast cancer screening: An independent review. Lancet.

[5] Bleyer A., Welch H.G. (2012). Effect of Three Decades of Screening Mammography on Breast-Cancer Incidence. N. Engl. J. Med..

[6] Mavaddat N., Michailidou K., Dennis J., Lush M., Fachal L., Lee A., Tyrer J.P., Chen T.-H., Wang Q., Bolla M.K. (2019). Polygenic Risk Scores for Prediction of Breast Cancer and Breast Cancer Subtypes. Am. J. Hum. Genet..

[7] Sokolova A., Johnstone K.J., McCart Reed A.E., Simpson P.T., Lakhani S.R. (2022). Hereditary breast cancer: Syndromes, tumour pathology and molecular testing. Histopathology.

[8] Quante A.S., Engel C., Kiechle M., Schmutzler R.K., Fischer C. (2020). Changes in risk calculation for the intensified surveillance programme of the German Consortium for Breast and Ovarian Cancer. Gynakologe.

[9] Kuchenbaecker K.B., Hopper J.L., Barnes D.R., Phillips K.-A., Mooij T.M., Roos-Blom M.-J., Jervis S., Van Leeuwen F.E., Milne R.L., Andrieu N. (2017). Risks of breast, ovarian, and contralateral breast cancer for BRCA1 and BRCA2 mutation carriers. JAMA—J. Am. Med. Assoc..

[10] Schon K., Tischkowitz M. (2018). Clinical implications of germline mutations in breast cancer: TP53. Breast Cancer Res. Treat..

[11] Antoniou A.C., Casadei S., Heikkinen T., Barrowdale D., Pylkäs K., Roberts J., Lee A., Subramanian D., De Leeneer K., Fostira F. (2014). Breast-Cancer Risk in Families with Mutations in PALB2. N. Engl. J. Med..

[12] Easton D.F., Pharoah P.D., Antoniou A.C., Tischkowitz M., Tavtigian S.V., Nathanson K.L., Devilee P., Meindl A., Couch F.J., Southey M. (2015). Gene-Panel Sequencing and the Prediction of Breast-Cancer Risk. N. Engl. J. Med..

[13] Mavaddat N., Pharoah P.D.P., Michailidou K., Tyrer J., Brook M.N., Bolla M.K., Wang Q., Dennis J., Dunning A.M., Shah M. (2015). Prediction of breast cancer risk based on profiling with common genetic variants. J. Natl. Cancer Inst..

[14] Vachon C.M., Pankratz V.S., Scott C.G., Haeberle L., Ziv E., Jensen M.R., Brandt K.R., Whaley D.H., Olson J.E., Heusinger K. (2015). The contributions of breast density and common genetic variation to breast cancer risk. J. Natl. Cancer Inst..

[15] Bahcall O.G. (2013). ICOGS collection provides a collaborative model. Nat. Genet..

[16] Hall P., Easton D. (2013). Breast cancer screening: Time to target women at risk. Br. J. Cancer.

[17] Kuchenbaecker K.B., McGuffog L., Barrowdale D., Lee A., Soucy P., Dennis J., Domchek S.M., Robson M., Spurdle A.B., Ramus S.J. (2017). Evaluation of polygenic risk scores for breast and ovarian cancer risk prediction in BRCA1 and BRCA2 mutation carriers. J. Natl. Cancer Inst..

[18] Gallagher S., Hughes E., Wagner S., Tshiaba P., Rosenthal E., Roa B.B., Kurian A.W., Domchek S.M., Garber J., Lancaster J. (2020). Association of a Polygenic Risk Score with Breast Cancer among Women Carriers of High- And Moderate-Risk Breast Cancer Genes. JAMA Netw. Open.

[19] Rouge-Bugat M.-E., Balleyguier C., Laurent N., Dautreppe A., Maillet L., Simon P., Fournet P., Scellier C., Menini T., Darmon E. (2022). MyPeBS International randomized study comparing personalised, risk-stratified to standard breast cancer screening in women aged 40–70: Focus on recruitment strategy in France. Presse Médicale Open.

[20] Louro J., Posso M., Boon M.H., Román M., Domingo L., Castells X., Sala M. (2019). A systematic review and quality assessment of individualised breast cancer risk prediction models. Br. J. Cancer.

[21] Lee A., Mavaddat N., Wilcox A.N., Msc A.P.C., Carver T., Hartley S., de Villiers C.B., Izquierdo A., Simard J., Schmidt M.K. (2019). BOADICEA: A comprehensive breast cancer risk prediction model incorporating genetic and nongenetic risk factors. Genet. Med..

[22] Choudhury P.P., Brook M.N., Hurson A.N., Lee A., Mulder C.V., Coulson P., Schoemaker M.J., Jones M.E., Swerdlow A.J., Chatterjee N. (2021). Comparative validation of the BOADICEA and Tyrer-Cuzick breast cancer risk models incorporating classical risk factors and polygenic risk in a population-based prospective cohort of women of European ancestry. Breast Cancer Res..

[23] Yang X., Eriksson M., Czene K., Lee A., Leslie G., Lush M., Wang J., Dennis J., Dorling L., Carvalho S. (2022). Prospective validation of the BOADICEA multifactorial breast cancer risk prediction model in a large prospective cohort study. J. Med. Genet..

[24] Lakeman I.M.M., Rodríguez-Girondo M., Lee A., Ruiter R., Stricker B.H., Wijnant S.R.A., Kavousi M., Antoniou A.C., Schmidt M.K., Uitterlinden A.G. (2020). Validation of the BOADICEA model and a 313-variant polygenic risk score for breast cancer risk prediction in a Dutch prospective cohort. Genet. Med..

[25] Shieh Y., Hu D., Ma L., Huntsman S., Gard C.C., Leung J.W.T., Tice J.A., Vachon C.M., Cummings S.R., Kerlikowske K. (2016). Breast cancer risk prediction using a clinical risk model and polygenic risk score. Breast Cancer Res. Treat..

[26] Dite G.S., MacInnis R.J., Bickerstaffe A., Dowty J.G., Allman R., Apicella C., Milne R.L., Tsimiklis H., Phillips K.-A., Giles G.G. (2016). Breast cancer risk prediction using clinical models and 77 independent risk-associated SNPs for women aged under 50 years: Australian breast cancer family registry. Cancer Epidemiol. Biomark. Prev..

[27] Maas P., Barrdahl M., Joshi A.D., Auer P.L., Gaudet M.M., Milne R.L., Schumacher F.R., Anderson W.F., Check D., Chattopadhyay S. (2016). Breast Cancer Risk From Modifiable and Nonmodifiable Risk Factors Among White Women in the United States. JAMA Oncol..

[28] Zhang X., Rice M., Tworoger S.S., Rosner B.A., Eliassen A.H., Tamimi R.M., Joshi A.D., Lindstrom S., Qian J., Colditz G.A. (2018). Addition of a polygenic risk score, mammographic density, and endogenous hormones to existing breast cancer risk prediction models: A nested case–control study. PLoS Med..

[29] TCarver T., Hartley S., Lee A., Cunningham A.P., Archer S., de Villiers C.B., Roberts J., Ruston R., Walter F.M., Tischkowitz M. (2021). Canrisk tool—A web interface for the prediction of breast and ovarian cancer risk and the likelihood of carrying genetic pathogenic variants. Cancer Epidemiol. Biomark. Prev..

[30] Archer S., De Villiers C.B., Scheibl F., Carver T., Hartley S., Lee A., Cunningham A.P., Easton D.F., McIntosh J.G., Emery J. (2020). Evaluating clinician acceptability of the prototype CanRisk tool for predicting risk of breast and ovarian cancer: A multi-methods study. PLoS ONE.

[31] Mavaddat N., Ficorella L., Carver T., Lee A., Cunningham A.P., Lush M., Dennis J., Tischkowitz M., Downes K., Hu D. (2023). Incorporating Alternative Polygenic Risk Scores into the BOADICEA Breast Cancer Risk Prediction Model. Cancer Epidemiol. Biomark. Prev..

[32] CanRisk Knowledge Base: Risk Calculations. https://canrisk.atlassian.net/wiki/spaces/FAQS/pages/131203073/Why+has+the+program+calculated+mutation+carrier+probabilities+but+not+cancer+risks.

[33] Girdea M., Dumitriu S., Fiume M., Bowdin S., Boycott K.M., Chénier S., Chitayat D., Faghfoury H., Meyn M.S., Ray P.N. (2013). PhenoTips: Patient phenotyping software for clinical and research use. Hum. Mutat..

[34] Deutsche Krebsgesellschaft Checkliste zur Erfassung einer familiären Belastung für Brust- und Eierstockkrebs. https://www.krebsgesellschaft.de/zertdokumente.html.

[35] Rhiem K., Bücker-Nott H., Hellmich M., Fischer H., Ataseven B., Dittmer-Grabowski C., Latos K., Pelzer V., Seifert M., Schmidt A. (2019). Benchmarking of a checklist for the identification of familial risk for breast and ovarian cancers in a prospective cohort. Breast J..

[36] Kast K., Rhiem K., Wappenschmidt B., Hahnen E., Hauke J., Bluemcke B., Zarghooni V., Herold N., Ditsch N., Kiechle M. (2016). Prevalence of *BRCA1/2* germline mutations in 21 401 families with breast and ovarian cancer. J. Med. Genet..

[37] Harter P., Hauke J., Heitz F., Reuss A., Kommoss S., Marmé F., Heimbach A., Prieske K., Richters L., Burges A. (2017). Prevalence of deleterious germline variants in risk genes including BRCA1/2 in consecutive ovarian cancer patients (AGO-TR-1). PLoS ONE.

[38] Engel C., Rhiem K., Hahnen E., Loibl S., Weber K.E., Seiler S., Zachariae S., Hauke J., Wappenschmidt B., Waha A. (2018). Prevalence of pathogenic BRCA1/2 germline mutations among 802 women with unilateral triple-negative breast cancer without family cancer history. BMC Cancer.

[39] Richards S., Aziz N., Bale S., Bick D., Das S., Gastier-Foster J., Grody W.W., Hegde M., Lyon E., Spector E. (2015). Standards and guidelines for the interpretation of sequence variants: A joint consensus recommendation of the American College of Medical Genetics and Genomics and the Association for Molecular Pathology. Genet. Med..

[40] Landrum M.J., Lee J.M., Riley G.R., Jang W., Rubinstein W.S., Church D.M., Maglott D.R. (2014). ClinVar: Public archive of relationships among sequence variation and human phenotype. Nucleic Acids Res..

[41] Stenson P.D., Ball E.V., Mort M., Phillips A.D., Shiel J.A., Thomas N.S., Abeysinghe S., Krawczak M., Cooper D.N. (2003). Human Gene Mutation Database (HGMD^®^): 2003 Update. Human. Mutat..

[42] Stenson P.D., Ball E.V., Mort M., Phillips A.D., Shiel J.A., Thomas N.S., Abeysinghe S., Krawczak M., Cooper D.N. (2021). HerediCaRe: Documentation and IT Solution of a Specialized Registry for Hereditary Breast and Ovarian Cancer. Gesundheitswesen Suppl..

[43] Stenson P.D., Ball E.V., Mort M., Phillips A.D., Shiel J.A., Thomas N.S., Abeysinghe S., Krawczak M., Cooper D.N. (2020). The mutational constraint spectrum quantified from variation in 141,456 humans. Nature.

[44] Kircher M., Witten D.M., Jain P., O’roak B.J., Cooper G.M., Shendure J. (2019). A general framework for estimating the relative pathogenicity of human genetic variants. Nat. Genet..

[45] Jaganathan K., Panagiotopoulou S.K., McRae J.F., Darbandi S.F., Knowles D., Li Y.I., Kosmicki J.A., Arbelaez J., Cui W., Schwartz G.B. (2019). Predicting Splicing from Primary Sequence with Deep Learning. Cell.

[46] Babraham Bioinformatics FastQC. https://www.bioinformatics.babraham.ac.uk/projects/fastqc/.

[47] Bolger A.M., Lohse M., Usadel B. (2014). Usadel Trimmomatic: A flexible trimmer for Illumina sequence data. Bioinformatics.

[48] Li H. (2018). Minimap2: Pairwise alignment for nucleotide sequences. Bioinformatics.

[49] Li H., Handsaker B., Wysoker A., Fennell T., Ruan J., Homer N., Marth G., Abecasis G., Durbin R., 1000 Genome Project Data Processing Subgroup (2009). The Sequence Alignment/Map format and SAMtools. Bioinformatics.

[50] Garrison E., Marth G. (2012). Haplotype-Based Variant Detection from Short-read Sequencing. http://arxiv.org/abs/1207.3907.

[51] Virtanen P., Gommers R., Oliphant T.E., Haberland M., Reddy T., Cournapeau D., Burovski E., Peterson P., Weckesser W., Bright J. (2020). SciPy 1.0: Fundamental algorithms for scientific computing in Python. Nat. Methods.

[52] CanRisk Knowledge Base: What Variants are Used in the PRS?. https://canrisk.atlassian.net/wiki/spaces/FAQS/pages/35979266/What+variants+are+used+in+the+PRS.

[53] Rhiem K., Auber B., Briest S., Dikow N., Ditsch N., Dragicevic N., Grill S., Hahnen E., Horvath J., Jaeger B. (2022). Consensus Recommendations of the German Consortium for Hereditary Breast and Ovarian Cancer. Breast Care.

[54] Bick U., Engel C., Krug B., Heindel W., Fallenberg E.M., Rhiem K., Maintz D., Golatta M., Speiser D., Rjosk-Dendorfer D. (2019). High-risk breast cancer surveillance with MRI: 10-year experience from the German consortium for hereditary breast and ovarian cancer. Breast Cancer Res. Treat..

[55] Waha A., Versmold B., Kast K., Kiechle M., Ditsch N., Meindl A., Niederacher D., Hahnen E., Arnold N., Mundhenke C. (2018). Konsensusempfehlung des Deutschen Konsortiums Familiärer Brust- und Eierstockkrebs zum Umgang mit Ergebnissen der Multigenanalyse. Tumor Diagn. Und Ther..

[56] (2017). IKNL Richtlijn Borstkanker—Screening Buiten het Bevolkingsonderzoek. https://richtlijnendatabase.nl/richtlijn/borstkanker/screening/screening_buiten_het_bob/screening_buiten_het_bevolkingsonderzoek.html.

[57] (2013). National Institute for Health and Care Excellence Familial Breast Cancer: Classification, Care and Managing Breast Cancer and Related Risks in People with a Family History of Breast Cancer Clinical Guideline Your Responsibility. www.nice.org.uk/guidance/cg164.

[58] NCCN (2017). Clinical Practice Guidelines in Oncology; Breast Cancer Screening and Diagnosis. https://www.nccn.org/professionals/physician_gls/pdf/breast-screening.pdf.

[59] Lakeman I.M.M., Rodríguez-Girondo M.D.M., Lee A., Celosse N., Braspenning M.E., van Engelen K., van de Beek I., van der Hout A.H., García E.B.G., Mensenkamp A.R. (2022). Clinical applicability of the Polygenic Risk Score for breast cancer risk prediction in familial cases Cancer genetics. J. Med. Genet..

[60] Michailidou K., Lindstrom S., Dennis J., Beesley J., Hui S., Kar S., Lemacon A., Soucy P., Glubb D., Rostamianfar A. (2017). Association analysis identifies 65 new breast cancer risk loci. Nature.

[61] Adedokun B., Du Z., Gao G., Ahearn T.U., Lunetta K.L., Zirpoli G., Figueroa J., John E.M., Bernstein L., Zheng W. (2021). Cross-ancestry GWAS meta-analysis identifies six breast cancer loci in African and European ancestry women. Nat. Commun..

[62] Zhang H., Ahearn T.U., Lecarpentier J., Barnes D., Beesley J., Qi G., Jiang X., O’mara T.A., Zhao N., kConFab Investigators (2020). Genome-wide association study identifies 32 novel breast cancer susceptibility loci from overall and subtype-specific analyses. Nat. Genet..

[63] Dixon P., Keeney E., Taylor J.C., Wordsworth S., Martin R.M. (2022). Can polygenic risk scores contribute to cost-effective cancer screening? A systematic review. Genet. Med..

[64] Ho W.-K., Tan M.-M., Mavaddat N., Tai M.-C., Mariapun S., Li J., Ho P.-J., Dennis J., Tyrer J.P., Bolla M.K. (2020). European polygenic risk score for prediction of breast cancer shows similar performance in Asian women. Nat. Commun..

[65] Shieh Y., Fejerman L., Lott P.C., Marker K., Sawyer S.D., Hu D., Huntsman S., Torres J., Echeverry M., E Bohórquez M. (2020). A Polygenic Risk Score for Breast Cancer in US Latinas and Latin American Women. J. Natl. Cancer Inst..

