# Supplementary material for: Clinical Impact of Polygenic Risk Score for Breast Cancer Risk Prediction in 382 Individuals with Hereditary Breast and Ovarian Cancer Syndrome
Source: Cancers (Basel). 2023 Aug 2;15(15):3938. doi: 10.3390/cancers15153938 (PMC10417109; doi:10.3390/cancers15153938)
Supplement: Supplementary file 1 [file cancers-15-03938-s001.zip › cancers-2492516-supplementary.pdf]

Supplementary materials

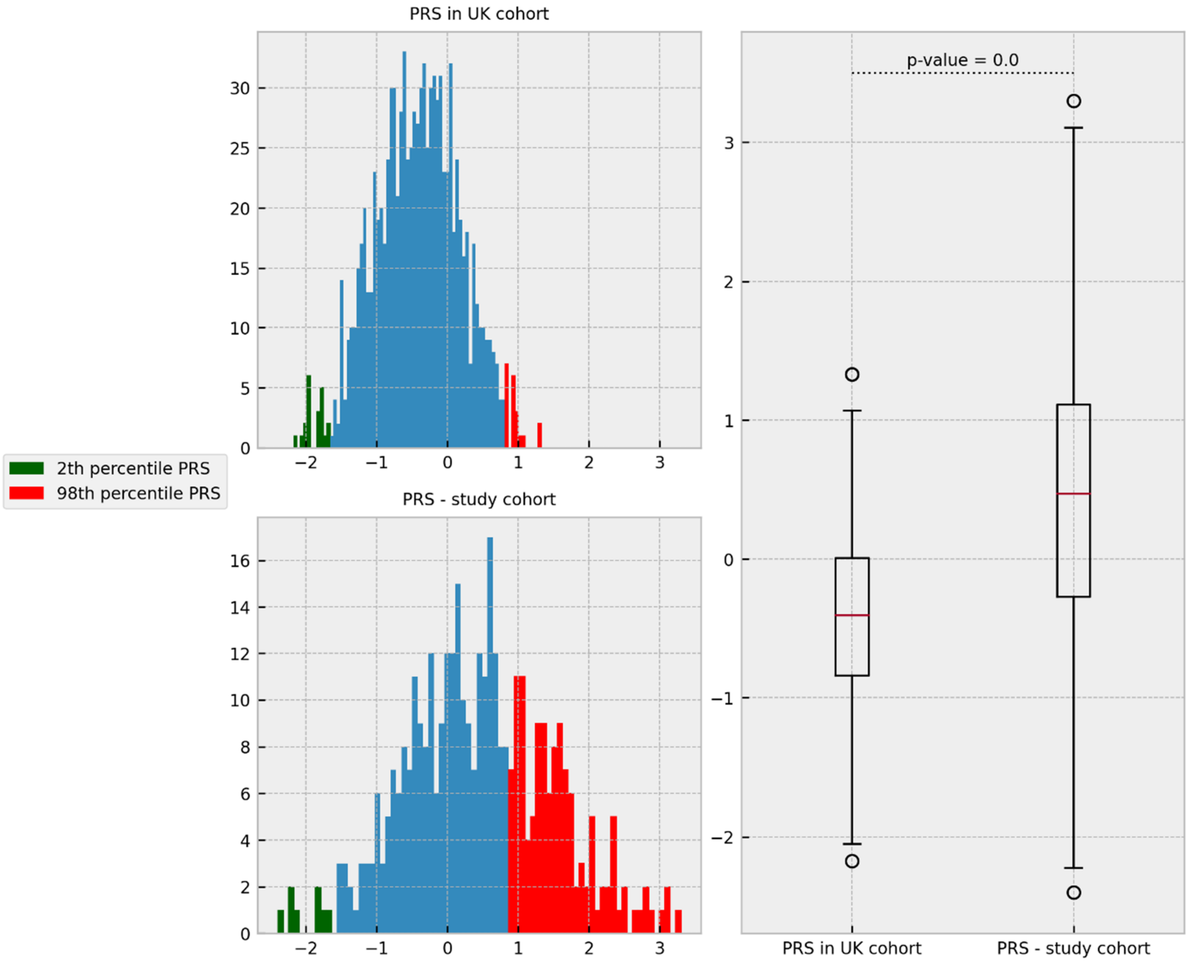

Supplemental Figure S1 The distribution of the PRS in our cohort and that of the UK cohort. The PRS is normally distributed; PRS = polygenic risk score

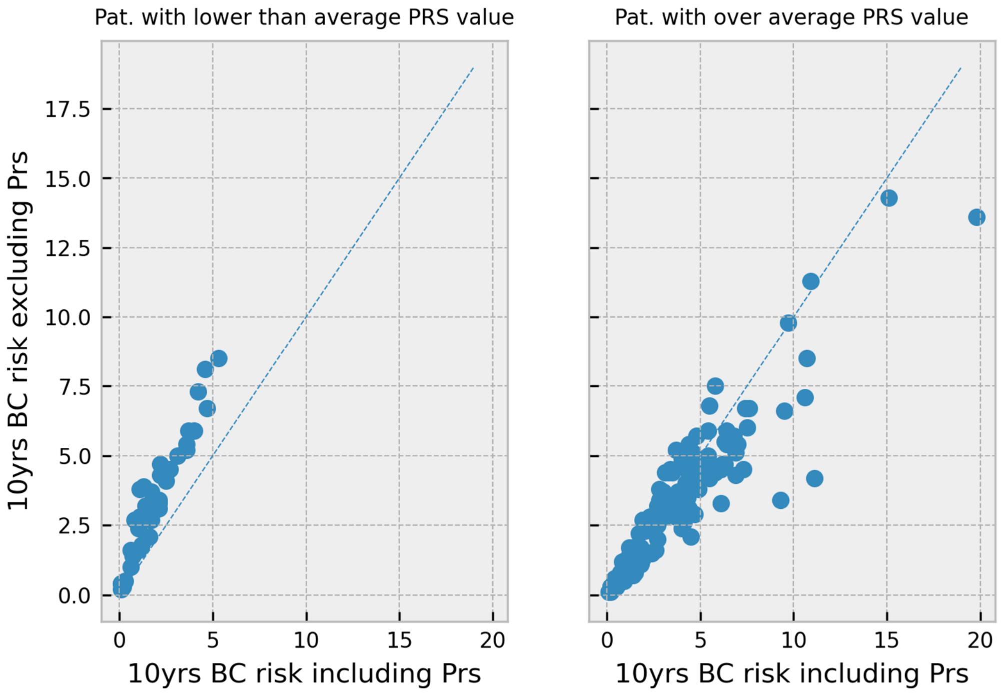

Supplemental Figure S2 Scatter plot of the change in individual breast cancer 10-year risk after inclusion of the PRS for all BC cases under the age of 50. Individuals are separated into high and low PRS values compared to the mean PRS value described for the BCAC-313 model; BC = breast cancer, BCAC=Breast Cancer Association Consortium, PRS = polygenic risk score; PV = pathogenic variant
